# Supplementary material for: The Systems Biology Research Tool: evolvable open-source software
Source: BMC Syst Biol. 2008 Jun 29;2:55. doi: 10.1186/1752-0509-2-55 (PMC2446383; doi:10.1186/1752-0509-2-55)
Supplement: Additional file 1 — SBRT Archive. An archive of the current version of the Systems Biology Research Tool. [file 1752-0509-2-55-S1.zip › sbrt-1.4.0/doc/developers_guide/api/sbrt/shell/io/fba/FluxCapInputFile.html]

FluxCapInputFile


|  |  |  |
| --- | --- | --- |
| **Overview** \| **Package** \| **Class** \| **Use** \| **Tree** \| **Deprecated** \| **Index** \| **Help** | |  |
| **PREV CLASS**   **NEXT CLASS** | **FRAMES**    **NO FRAMES**     **All Classes** |  |
| SUMMARY: NESTED \| FIELD \| CONSTR \| METHOD | DETAIL: FIELD \| CONSTR \| METHOD |  |


---


## sbrt.shell.io.fba Class FluxCapInputFile

```
java.lang.Object
  sbrt.shell.io.fba.FluxCapInputFile
```

**All Implemented Interfaces:**: java.lang.Iterable<java.util.Map<java.lang.String,java.util.Set<LinearComb<java.lang.String>>>>, InputFile<java.util.Map<java.lang.String,java.util.Set<LinearComb<java.lang.String>>>>, MapInputFile<java.lang.String,java.util.Set<LinearComb<java.lang.String>>>, MapTextFile<java.lang.String,java.util.Set<LinearComb<java.lang.String>>>, MapTextInputFile<java.lang.String,java.util.Set<LinearComb<java.lang.String>>>, SbrtFile

---

``` public class FluxCapInputFile extends java.lang.Object implements MapTextInputFile<java.lang.String,java.util.Set<LinearComb<java.lang.String>>> ```

This class is used to read files containing flux caps.
Flux caps are used in Mahadevan-Schilling flux range experiments.

**Author:**
:   This class was written and documented by
    Jeremiah Wright while in the Wagner lab.

---

**Constructor Summary**

`FluxCapInputFile(java.lang.String fileName,
FluxCapFormat lineFormat)`
  
          Constructs a new flux cap input file.


| **Method Summary** | |
| --- | --- |
| `void` | `close()`             Closes this file. |
| `java.lang.String` | `getCanonicalName()`             Returns the canonical name of this file. |
| `UnmodifiableMap<java.lang.String,java.util.Set<LinearComb<java.lang.String>>>` | `getFluxCaps()`             Returns the flux caps contained in this file. |
| `Fluxome` | `getFluxome()`             Returns the fluxome used to verify reaction names. |
| `MapFormat<java.lang.String,java.util.Set<LinearComb<java.lang.String>>>` | `getMapFormat()`             Returns the map format used to parse this file. |
| `java.lang.String` | `getOriginalName()`             Returns the name of this file. |
| `boolean` | `isClosed()`             Indicates if this file has already been closed. |
| `java.util.Iterator<java.util.Map<java.lang.String,java.util.Set<LinearComb<java.lang.String>>>>` | `iterator()`             Returns an iterator over this file. |

**Methods inherited from class java.lang.Object**

`clone, equals, finalize, getClass, hashCode, notify, notifyAll, toString, wait, wait, wait`

**Constructor Detail**

### FluxCapInputFile

```
public FluxCapInputFile(java.lang.String fileName,
                        FluxCapFormat lineFormat)
```

:   Constructs a new flux cap input file.

    **Parameters:**: `fileName` - the name of the file to read.: `lineFormat` - the format used to parse the data strings in this file.


**Method Detail**

### getFluxome

```
public Fluxome getFluxome()
```

:   Returns the fluxome used to verify reaction names.

    :   **Returns:**: the fluxome used to verify reaction names.

---


### getFluxCaps

```
public UnmodifiableMap<java.lang.String,java.util.Set<LinearComb<java.lang.String>>> getFluxCaps()
```

:   Returns the flux caps contained in this file.

    :   **Returns:**: the flux caps contained in this file.

---


### getMapFormat

```
public MapFormat<java.lang.String,java.util.Set<LinearComb<java.lang.String>>> getMapFormat()
```

:   Returns the map format used to parse this file.

    :   **Specified by:**: `getMapFormat` in interface `MapTextFile<java.lang.String,java.util.Set<LinearComb<java.lang.String>>>`
    :   **Returns:**: the map format used to parse this file.

---


### close

```
public void close()
```

:   Closes this file. If this file is already closed, then
    invoking this method has no effect.

    :   **Specified by:**: `close` in interface `SbrtFile`

---


### getOriginalName

```
public final java.lang.String getOriginalName()
```

:   Returns the name of this file.

    :   **Specified by:**: `getOriginalName` in interface `SbrtFile`
    :   **Returns:**: the name of this file.

---


### getCanonicalName

```
public final java.lang.String getCanonicalName()
```

:   Returns the canonical name of this file.

    :   **Specified by:**: `getCanonicalName` in interface `SbrtFile`
    :   **Returns:**: the canonical name of this file. **See Also:**: `File.getCanonicalPath()`

---


### isClosed

```
public boolean isClosed()
```

:   Indicates if this file has already been closed.

    :   **Specified by:**: `isClosed` in interface `SbrtFile`
    :   **Returns:**: `true` if this file is closed; `false` othewise.

---


### iterator

```
public java.util.Iterator<java.util.Map<java.lang.String,java.util.Set<LinearComb<java.lang.String>>>> iterator()
```

:   Returns an iterator over this file. This iterator
    returns only a single map.

    :   **Specified by:**: `iterator` in interface `java.lang.Iterable<java.util.Map<java.lang.String,java.util.Set<LinearComb<java.lang.String>>>>`
    :   **Returns:**: an iterator over this file. **See Also:**: `getFluxCaps()`


---


|  |  |  |
| --- | --- | --- |
| **Overview** \| **Package** \| **Class** \| **Use** \| **Tree** \| **Deprecated** \| **Index** \| **Help** | |  |
| **PREV CLASS**   **NEXT CLASS** | **FRAMES**    **NO FRAMES**     **All Classes** |  |
| SUMMARY: NESTED \| FIELD \| CONSTR \| METHOD | DETAIL: FIELD \| CONSTR \| METHOD |  |


---
